# Estimation of breeding population size using DNA‐based pedigree reconstruction in brown bears

**DOI:** 10.1002/ece3.9246

**Published:** 2022-09-06

**Authors:** Michito Shimozuru, Mina Jimbo, Keisuke Adachi, Kei Kawamura, Yuri Shirane, Yoshihiro Umemura, Tsuyoshi Ishinazaka, Masanao Nakanishi, Mayu Kiyonari, Masami Yamanaka, Yukihiro Amagai, Ayaho Ijuin, Tomoki Sakiyama, Shinsuke Kasai, Takane Nose, Masataka Shirayanagi, Hifumi Tsuruga, Tsutomu Mano, Toshio Tsubota, Keita Fukasawa, Hiroyuki Uno

**Affiliations:** ^1^ Laboratory of Wildlife Biology and Medicine, Faculty of Veterinary Medicine Hokkaido University Sapporo Japan; ^2^ Hokkaido Research Organization Sapporo Japan; ^3^ Shiretoko Nature Foundation Shari Japan; ^4^ Center for Environmental Biology and Ecosystem Studies National Institute for Environmental Studies Tsukuba Japan; ^5^ Faculty of Agriculture Tokyo University of Agriculture and Technology Tokyo Japan

**Keywords:** breeding population size, brown bear, microsatellite analysis, pedigree reconstruction, population estimate, *Ursus arctos*

## Abstract

Robust estimates of demographic parameters are critical for effective wildlife conservation and management but are difficult to obtain for elusive species. We estimated the breeding and adult population sizes, as well as the minimum population size, in a high‐density brown bear population on the Shiretoko Peninsula, in Hokkaido, Japan, using DNA‐based pedigree reconstruction. A total of 1288 individuals, collected in and around the Shiretoko Peninsula between 1998 and 2020, were genotyped at 21 microsatellite loci. Among them, 499 individuals were identified by intensive genetic sampling conducted in two consecutive years (2019 and 2020) mainly by noninvasive methods (e.g., hair and fecal DNA). Among them, both parents were assigned for 330 bears, and either maternity or paternity was assigned to 47 and 76 individuals, respectively. The subsequent pedigree reconstruction indicated a range of breeding and adult (≥4 years old) population sizes: 128–173 for female breeders and 66–91 male breeders, and 155–200 for female adults and 84–109 male adults. The minimum population size was estimated to be 449 (252 females and 197 males) in 2019. Long‐term continuous genetic sampling prior to a short‐term intensive survey would enable parentage to be identified in a population with a high probability, thus enabling reliable estimates of breeding population size for elusive species.

## Graphical abstract

This study estimated the breeding and adult population sizes, as well as the minimum population size, in a high‐density brown bear population on the Shiretoko Peninsula, in Hokkaido, Japan, using DNA‐based pedigree reconstruction. This study showed that combination of a short‐term intensive genetic survey and long‐term continuous genetic sampling prior to it enables parentage to be identified in a population with a high probability, thus enabling reliable estimates of breeding population size for elusive species.

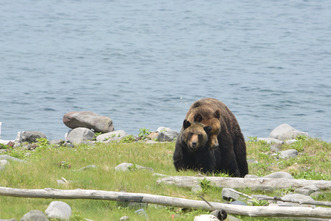

## 1 INTRODUCTION

The identification of demographic parameters is fundamental for understanding behavioral ecology (Roy et al., 2012; Stoen et al., 2006) and is essential for the effective management and conservation of wild animals (Katzner et al., 2011). This includes quantity‐related factors, such as population size/density and the number of reproductively active individuals, and quality‐related factors, such as sex ratios, age structures, survival/mortality rates, reproductive rate, and population growth rate. Reliable estimates of these reproductive parameters are of particular importance for endangered/overabundant animals or populations. These are critical for efficient detection of population declines or increases and to determine their causes, which are necessary to allow quick and precise actions, for example, hunting bans (Rosenblatt et al., 2014) and implementation of population control measures (Ueno et al., 2010). They are also essential to evaluate the future risk of extinction and recovery strategies for endangered species/populations (Haines et al., 2005).

However, it is usually challenging to obtain robust estimates of demographic parameters; this is particularly true for rare or elusive species, including large carnivores, most of which have declining population trends (Wolf & Ripple, 2018). In addition to habitat loss and fragmentation by deforestation (Zemanova et al., 2017), human‐caused mortality, including culling for management purposes and hunting have become a serious threat to populations (Collins & Kays, 2011). On the contrary, an increase in the population of large carnivores potentially enhances human–wildlife conflict (Hristienko & McDonald, 2007). Therefore, population monitoring of wild carnivores inhabiting areas close to human populations is indispensable for the development of wildlife management and conservation policies, such as determining harvest quotas (Kohira et al., 2009; Swenson et al., 1994), and also for taking immediate actions to mitigate human–wildlife conflict.

In the last two decades, DNA‐based statistical models have been developed and used to estimate population sizes and trends. Most are based on noninvasive sampling methods. In large carnivore studies, this includes the collection of hair (Rounsville et al., 2022; Woods et al., 1999), feces (Kindberg et al., 2011; Kohn et al., 1999), and their combination (Ciucci et al., 2015). Hair and fecal samples allow DNA‐based individual identification without capturing and handling the animals, which is of great advantage in terms of cost‐effectiveness (Kindberg et al., 2011), and animal welfare (Cattet et al., 2008). Several estimators have been developed for population size estimation based on noninvasive genetic data, including capture–mark–recapture (CMR) methods (Seber, 1986), rarefaction analysis (Kohn et al., 1999), and, more recently, spatially explicit capture–recapture (SECR) methods (Efford, 2004). These methods have been applied to several large carnivore species, including brown bears (*Ursus arctos*) (Bischof et al., 2020; Kindberg et al., 2011; Morehouse & Boyce, 2016), wolves (*Canis lupus*) (Caniglia et al., 2012), coyotes (*Canis latrans*) (Kohn et al., 1999; Morin et al., 2016), and mountain lions (*Puma concolor*) (Russell et al., 2012). These methods use an individual's genotype as a molecular tag (Schwartz et al., 2007). However, genotypic data are more than just tags; they contain further information, which can sometimes enable the analysis of parent–offspring relationships and improve the accuracy of estimates of population sizes and trends (Pearse et al., 2001).

As an alternative method for estimating demographic parameters, a DNA‐based pedigree reconstruction approach has been developed (Creel & Rosenblatt, 2013). This approach has been widely used to estimate the number of breeding individuals in a population (Israel & May, 2010; Koch et al., 2008; Pearse et al., 2001; Quinn et al., 2019), as well as to investigate many aspects of animal behavior, including population structure (Calboli et al., 2008; Hudy et al., 2010), breeding ecology (Levine et al., 2019; Shimozuru et al., 2019), and dispersal (Arora et al., 2012). Because population estimations based on statistical models do not provide age‐related information, estimates of breeding population size (generally defined as the number of females/males producing/siring offspring in a given period) can provide more practical information regarding the reproductive potential of a population. One of the advantages of this method is that it enables the inclusion of breeders that were not directly sampled to be inferred if their offspring have been sampled. However, it remains uncertain whether they were dead or alive at the time of sampling. Therefore, this method is particularly useful for estimating the number of breeding individuals under the circumstances where the inferred breeders can be determined to be alive or dead. For example, in a previous study in painted turtles (*Chrysemys picta*), Pearse et al. (2001) targeted hatchlings as offspring in a candidate parentage analysis, in addition to their mothers attending the nest, which enabled them to determine the number of male breeders that existed at the copulating period. In most mammals, it is not possible to selectively sample newborns. In addition, it is almost impossible to obtain information on age by noninvasive genetic sampling, which makes it more difficult to know whether the breeders inferred by pedigree reconstruction are dead or alive. Such uncertainty over the survival/mortality of the breeders raises the ceiling of the maximum estimates and thereby impairs its accuracy. This holds particularly true for large carnivores that are relatively long‐lived, for which multiple generations can coexist in a population, and mortality is difficult to detect. Therefore, studies of breeding populations based on the pedigree reconstruction approach are challenging and remain rare for large carnivore populations (Creel & Rosenblatt, 2013; Spitzer et al., 2016).

**FIGURE 1:**
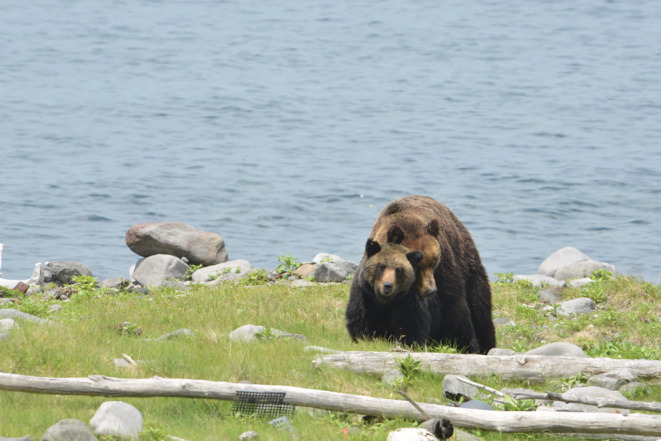
Brown bears copulating in the Rusha area of the Shiretoko peninsula, Hokkaido, Japan. (the photo was taken by Masami Yamanaka on June 24, 2018).

**FIGURE 2:**
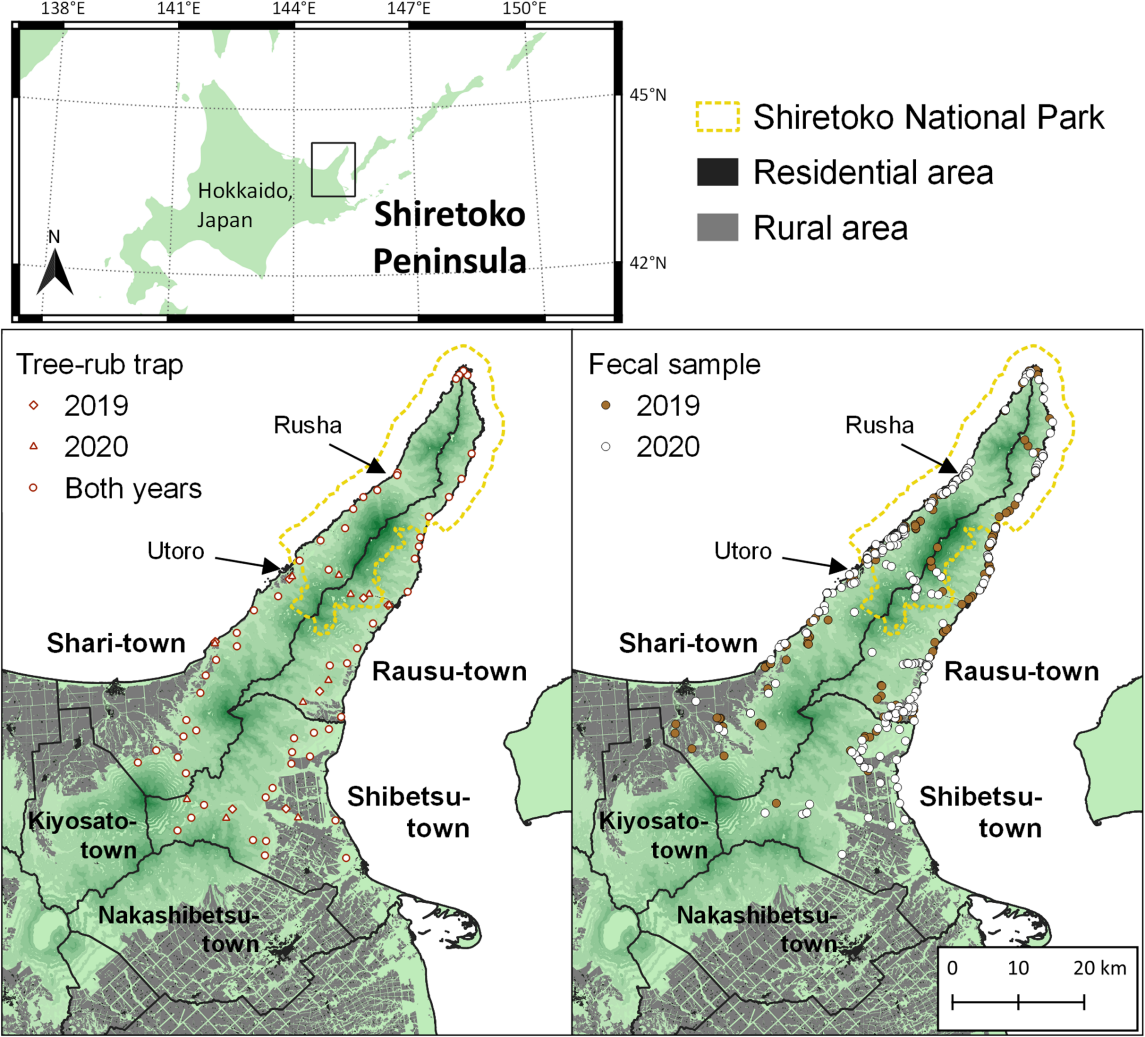
Map of the Shiretoko peninsula, eastern Hokkaido, Japan. The bottom‐left indicates the locations of tree‐rub traps installed in 2019–2020. The bottom‐right indicates the locations of fecal samples collected and successfully genotyped in 2019–2020. The dotted yellow line indicates the Shiretoko National Park. This map was created using QGIS version 3.4.7‐Madeira (QGIS.org, 2022. QGIS geographic information system. QGIS association. http://www.qgis.org) and edited by the author. The topographic features are based on digital topographic map 1:25,000 published by geospatial information Authority of Japan (available from https://fgd.gsi.go.jp/download/mapGis.php?tab=dem, accessed 18‐May‐2019). Administrative divisions were created by the National Land Numerical Information published by Ministry of Land, infrastructure, transport, and tourism of Japan (available from https://nlftp.mlit.go.jp/ksj/index.html, accessed 21‐Jul‐2021). National park boundaries were created using GIS data for national parks published by biodiversity center, Ministry of the Environment (available from http://gis.biodic.go.jp/webgis/sc‐026.html?kind=nps, 20‐Jul‐2021). The vegetation was created using the 1:25,000 GIS‐based vegetation map “Hokkaido” published by biodiversity Center of Japan, Ministry of the Environment, Japan (available from http://gis.biodic.go.jp/webgis/sc‐025.html?kind=vg67, 19‐Jul‐2021).

In this study, we estimated the breeding and adult population size, as well as the minimum population size, in a brown bear (Figure 1) population in the Shiretoko Peninsula, Hokkaido, Japan, based on a pedigree reconstruction approach. Brown bears occur throughout Hokkaido and are present at an especially high density in the Shiretoko Peninsula (Ministry of the Environment, Government of Japan, 2017). This small peninsula consists of coastal area and precipitous mountains, and most of the area has limited accessibility, which makes it difficult to conduct a population estimation survey based on a systematic genetic sampling targeting all areas of the peninsula. As an alternative, a harvest‐based method, based on the mortality records of brown bears, has estimated a population size as 559, although the wide confidence intervals (±440; 95% CI) give little credibility to the estimates (Ministry of the Environment, Government of Japan, 2017). The precise estimation of the population and/or breeding population would be required for the appropriate management and conservation of brown bears. On the peninsula, information on genotypes, sex, and ages of dead bears (due to management culls, hunting, accidents, or natural causes) has been collected for the past three decades. In addition, opportunistic noninvasive genetic sampling (hairs and feces) has been performed in some areas (Shirane et al., 2018), and continuous bear monitoring surveys (including DNA sampling) have been conducted for a decade or more in the Rusha area (Figure 2; Shimozuru et al., 2017). Under these conditions, the accumulated information, if combined with large‐scale, intensive genetic sampling (including hair‐traps and fecal collection), may be able to identify reliable demographic parameters in place of other methods (e.g., the CMR method, a rarefaction analysis, and the SECR method). In this study, we applied a pedigree reconstruction approach to this small but highly populated bear habitat. The population size of breeders and adults, and the minimum population size, were estimated based on large‐scale genetic sampling events conducted in two consecutive years.

## 2 METHODS

### 2.1 Ethics statement

All procedures involved in sample collection from live animals were conducted in accordance with the Guidelines for Animal Care and Use, Hokkaido University and were approved by the Animal Care and Use Committee of the Graduate School of Veterinary Medicine, Hokkaido University (Permit Number: 1106, 1151, 1152, 15,009, 17,005, 18–0083, and 19–0047).

### 2.2 Study area and sampling

This study was conducted on the Shiretoko Peninsula (43°50′–44°20′ N, 144°45′–145°20′ E; Figure 2), eastern Hokkaido, Japan. An area extending from the middle to the tip of the peninsula has been designated a UNESCO World Natural Heritage Site, as well as a national park, where the habitat of the brown bear is protected. However, human–bear conflict, including agricultural crop damage and intrusion into human residential areas, has become a serious problem on the peninsula. As many as 20–70 bears have been killed annually over the past decade, (total 373 bears in 2011–2020; the data were part of this study) mainly for management purposes. Biological samples were collected in and around the peninsula during 1998–2020 using multiple methods (i.e., hair‐traps, fecal collection, biopsy darting, tissue collection from bears killed due to nuisance control or hunting, and blood collection from bears captured for research purposes) that detailed in previous studies (Shimozuru et al., 2017, 2019; Shimozuru, Shirane, Jimbo, et al., 2020; Shimozuru, Shirane, Yamanaka, et al., 2020; Shirane et al., 2018, 2019). In this study, the area consisting of Shari, Rausu, and Shibetsu towns was defined as being inside the peninsula (approximately 1760 km^2^), with additional samples from Kiyosato and Nakashibetsu towns also included in the analysis (Figure 2). For bears captured or killed between 1998 and 2020, age was estimated by counting the dental cementum annuli (Craighead et al., 1970; Tochigi et al., 2018).

During 2019–2020, we conducted intensive, noninvasive genetic sampling for hair and feces. For hair, 63 and 67 tree‐rub traps (For details, see Sato et al., 2020; Shimozuru, Shirane, Jimbo, et al., 2020) were placed throughout the peninsula in 2019 and 2020, respectively, except for areas where it was difficult to gain access (Figure 2). In the tree‐rub trap, the trunk was partially smeared with wood preservative (Creosote R; Yoshida refinery, Tokyo, Japan) to lure bears (Sato et al., 2020), and barbed wire was wrapped around the trunk between 30 and 230 cm above the ground. From late May to October, we visited each trap at approximately 2‐week intervals (a total of 10 and 11 collections in 2019 and 2020, respectively), and collected hairs from individual barbs, which then were stored separately in envelopes. Samples were dried and kept at −30°C until DNA extraction. Each tree‐rub was monitored by an automatic camera (HykeCam SP108‐J; Hyke Inc., Asahikawa, Japan). The recording time and intervals were set to 25 and 5 s, respectively. All videos were checked to estimate the number of bears that potentially rubbed against the tree, and their sex/age status was determined visually if possible. Through a combination of genetic analysis and video data, breeding status was clarified in some females, for example, by the accompanying presence of cubs or yearlings. For fecal samples, we collected bear scats with ages of 0–4 days as estimated by field collectors. They were stored in Inhibitex buffer (Qiagen Inc., Tokyo, Japan) and kept at −30°C until DNA extraction. Bear scats were collected every time field collectors found them during bear patrols in and around popular tourist areas and farmland, driving on forest roads, and during exploratory investigations in the forest. To collect DNA samples from the areas without tree‐rub traps, field collectors periodically (1–2 times a month from June to September) made explorations on foot into those areas, for example, high‐elevation areas and the tip of the peninsula.

### 2.3 Extraction of DNA and genotyping

The extraction of DNA, microsatellite genotyping, and a mitochondrial DNA haplotype analysis were conducted using the procedure described in previous studies (Shimozuru et al., 2019; Shirane et al., 2018). Briefly, DNA was extracted using the DNeasy Blood & Tissue Mini Kit (Qiagen Inc., Tokyo, Japan) for blood and tissue samples, the DNA Extractor FM Kit (Wako, Osaka, Japan) or Isohair Easy (Nippon Gene, Inc., Tokyo, Japan) for hair samples, and the QIAamp DNA Stool Mini Kit (Qiagen Inc.) for feces samples. For hair and feces, each DNA sample was initially tested with two primer mixes including six loci (Primer A; G1A, MU05, and MU51, Primer B; MU50, G10B, and MU23; listed in Appendix A) as a screening step for individual identification. Our previous study showed that these six loci had sufficient power for individual discrimination, that is, the probability that two individuals share the same genotype was 1.3 × 10^−6^ (Shimozuru et al., 2019). If PCR amplification failed at any locus, the sample was excluded from further analyses. When a sample did not match precisely with any individual, it was tested with 21 microsatellite markers (Appendix A), including the above six markers and one sex marker, amelogenin (Yamamoto et al., 2002), by a multiplex PCR assay (Shimozuru et al., 2019; Shimozuru, Shirane, Jimbo, et al., 2020; Shimozuru, Shirane, Yamanaka, et al., 2020). Generally, the DNA quality was better in tissue/blood samples than in hair/feces and in hair than feces. If we obtained better quality samples from the same individual, all loci were tested again for confirmation. All individuals were genotyped for all loci two or more times. Allele size was determined using an ABI PRISM 310 genetic analyzer or SeqStudio Genetic Analyzer (Thermo Fisher Scientific K.K., Tokyo, Japan). In addition, eight Y‐linked microsatellite alleles (Y318.1, Y318.2, Y318.4, Y318.6, Y318.9, Y369.1, Y369.4, and 15020.1; Hirata et al., 2017) were determined by a multiplex PCR assay, using the same primer sets as those used in previous studies (Bidon et al., 2014; Hirata et al., 2017). The mitochondrial and Y chromosome haplotype information were used to select candidate mothers for offspring, and candidate fathers for male offspring, respectively.

### 2.4 Pedigree reconstruction

Parentage analysis was performed using a likelihood‐based approach with the CERVUS software (version 3.0.7) (Kalinowski et al., 2007), followed by the COLONY software (version 2.0.6.4) (Jones & Wang, 2010; Wang, 2004). We confirmed previously that all microsatellite loci can be included in parentage analysis, targeting this bear population, judging from the low null allele frequency (<0.05) and lack of significant deviation from Hardy–Weinberg equilibrium (Shimozuru et al., 2019; Shimozuru, Shirane, Jimbo, et al., 2020). Mitochondrial and Y chromosomal haplotype data were used for the selection of potential mother–offspring relationships and potential father–son relationships, respectively. Analyses were run systematically in accordance with a previous study (Shimozuru et al., 2019). First, all individuals, sampled during 1998–2020, were analyzed with CERVUS, which selected the most likely parent among the existing candidates. The same simulation parameters were set as in a previous study (Shimozuru et al., 2019; Shimozuru, Shirane, Yamanaka, et al., 2020). Briefly, the parameters included 10,000 cycles, 150 candidate mothers and fathers per offspring, 40% of candidate parents sampled, and 1% of loci mistyped. In the first step of the CERVUS analysis, we assigned a parent pair. The confidence level was set at 80%, and no allele mismatching in 21 microsatellite loci was allowed in a parent–offspring combination (i.e., mother–father–offspring trio). One mismatch was allowed in a parent–offspring combination obtained at a ≥ 95% confidence level when the same mother and father were selected as the most likely parents (≤1 mismatch per pair) in maternity and paternity assignment analyses, respectively. If a parent pair could not be assigned due to a low (<80%) confidence level or the presence of ≥1 mismatching loci, we assigned maternity or paternity as a second step. The confidence level was set at 80%, and no mismatching was allowed in a mother/father–offspring combination. Furthermore, bears that were not assigned a mother and/or father in CERVUS were included in a parentage analysis using COLONY. The COLONY software generates hypothetical parents in a sib‐ship reconstruction with the assumption that both females and males are promiscuous (Steyaert et al., 2012), which enables the assignment of parentage to individuals whose parent(s) were not present in the parent candidate data set. To reduce the possibility of multiple generations in the candidate offspring leading to a false parentage assignment, only bears that were sampled during 2019–2020 were included as candidate offspring in COLONY analyses. The same simulation parameters were set as in a previous study (Shimozuru et al., 2019). Briefly, we set the rate of the typing error and the additional error to 1%, according to CERVUS analysis. We allowed for male and female polygamy. The other parameters were set to the default settings: The length of the run was set to medium, the full likelihood method was adopted, and likelihood precision was set to medium.

### 2.5 Breeding/adult population estimates

In this study, breeders and adults were defined as bears that produced ≥1 offspring between 1998 and 2020, and bears that had the potential ability to breed, respectively. For females, adults included both breeders and bears ≥4 years old (the youngest age of the first mating in this population), reported by Shimozuru et al. (2017). For males, the youngest age of the first mating in this population was 6 years of age (Shimozuru, Shirane, Jimbo, et al., 2020), whereas males potentially reach sexual maturation at 3.5 years of age in the Scandinavian population (Zedrosser et al., 2007). In the current analysis, bears ≥4 years old and sexually experienced males (indicated by a parentage analysis) were included in the adult population, which allowed us to compare the breeding/adult population size between sexes. In this study, we estimated the breeding/adult population size as of 2019, the first year of the intensive genetic sampling period. This was because 2019 was expected to be the year when the highest number of breeders/adults would be identified as alive. For example, females identified with cubs for the first time in 2020 could be counted as breeders in 2019. Likewise, all females and males identified in 2019 and/or 2020 were confirmed as breeders if their offspring were sampled during 1998–2020. As breeders included females/males that had not produced any offspring as of 2019 in this study, the strict definition of breeders was females/males that had experienced mating that led to the production/siring of cubs in the following year. In terms of adult population size, we additionally included bears (≥4 years) whose birth year was identified in an ongoing bear monitoring survey that has been continuously conducted in recent decades in the area between Rusha and Utoro (Shimozuru et al., 2017; Shimozuru, Shirane, Yamanaka, et al., 2020). In addition, some bears were confirmed to be ≥4 years based on the year of first genetic identification, or on the year when their parent was dead (e.g., if the father was dead in 2014, his offspring could potentially be born in 2015, suggesting they were ≥4 years old as of 2019).

Unfortunately, no method has yet been described to assign confidence limits to breeding/adult population estimates based on pedigree reconstruction approaches (Creel & Rosenblatt, 2013; Spitzer et al., 2016), and we estimated the upper and lower bounds as follows. First, we calculated the minimum number of breeders, including the existing bears and hypothetical parents. The former included bears identified in 2019 and/or 2020 that were confirmed as reproductively successful based on a parentage analysis. The later included hypothetical parents generated by the COLONY software, which were estimated to produce cubs during 2018–2020. For example, if a 1‐year‐old bear, killed in 2019, was not assigned a mother from the list of candidate mothers, it was reasonable to assume that his/her mother, although not genetically identified, gave birth in 2018 and was alive until the timing of mother–offspring separation in 2019. Similarly, if a cub‐of‐the‐year, sampled in 2020, was not assigned a father from the list of candidate fathers, it could be assumed that his/her father was alive and mated with the mother in 2019.

Second, we estimated the maximum breeding population size by a pedigree reconstruction approach, based on the simple assumption that the number of breeders would not exceed the total number of parents that produced bears identified in 2019–2020. In addition to the minimum number of breeders, we included breeders (previously existed, but not identified in 2019–2020) that produced bears identified in 2019–2020, and hypothetical parents generated by COLONY software that were estimated to have produced bears (identified in 2019–2020) whose mother and/or father were missing from the list of existing candidates sampled during 1998–2020. In the former case, one problem of this approach is that it is difficult to know how many of the parents that were identified as alive until 2018, but not sampled during 2019–2020, were still alive as of 2019. To account for mortality among those individuals, we calculated the period between the year of the last identification and 2019, and multiplied it by the survival rate to estimate his/her survival probability. Because the adult survival rate was not investigated in this population, we applied the median value of the survival rates (0.94 for females and 0.89 for males) among the other brown bear populations (0.89–0.96 for females, 0.62–0.94 for males; reviewed in Schwartz, Miller, et al., 2003). For example, a mother identified as alive in 2017 was counted as 0.88 of an individual (i.e., 0.94 × 0.94). Another problem, in the latter case, is that the assumption that each missing parent constitutes a new individual would most likely cause an overestimation (Spitzer et al., 2016). In this study, COLONY allowed hypothetical parents to produce multiple offspring, which reduced the likelihood of overestimation due to this issue.

A similar but more serious concern, reported by Creel and Rosenblatt (2013), was that there is no way to ascertain how many of the hypothetical parents are actually alive. To avoid overestimation, we made several assumptions. First, females ≥30 years old and males ≥28 years old were not counted as breeding individuals. This assumption was based on previous studies regarding reproductive senescence in brown bears (Schwartz, Keating, et al., 2003; Van Daele et al., 2001; Zedrosser et al., 2007). Upon pedigree reconstruction, the age of each hypothetical parent was estimated based on the age of the oldest offspring and generation intervals. The generation interval between mother and offspring was set at 7.3 years based on our bear monitoring survey in the Rusha area. We calculated the first age when females gave birth to cubs that survived the first year (for eight females = 5–9 years, average of 7.25; Shimozuru et al., 2017), and used it as the minimum interval between generations. This value was more realistic than their primiparity age (for 15 females = 5–6 years, average of 5.3; Shimozuru et al., 2017), which was more likely to induce overestimation in the current analysis. The generation intervals between father and offspring, that is, the first age when males sired cubs that survived the first year, was not well investigated in this population. Males become sexually mature at 3.5 years old (Zedrosser et al., 2007), but it is rare to gain a reproductive opportunity until physical maturation at around 9–11 years of age (Moriwaki et al., 2018; Shimozuru, Shirane, Jimbo, et al., 2020; Shirane et al., 2020). Therefore, we set the same value (7.3 years) as for females, based on the assumption that the generation intervals between father and offspring were not less than those between mother and offspring. The second assumption was that more than four matrilineal generations do not exist at the same time, which was also based on our bear monitoring survey conducted in the Rusha area. In this area, four generations (offspring, mother, grandmother, and great‐grandmother) exist at the same time, but a great‐great‐grandmother has never been identified (Shimozuru et al., 2017). Similarly, our previous pedigree reconstruction conducted in the same population revealed that more than three paternal generations (offspring‐father‐grandfather) do not exist at the same time (Shimozuru et al., 2019). Hypothetical mothers and fathers that correspond to great‐great‐grandmother and great‐grandfather, respectively, were assumed to be dead, and were not counted as breeding individuals in the current analysis. Finally, we calculated the minimum and maximum number of adults, by adding the number of bears confirmed to be ≥4 years old in 2019 (based on the criteria described above) to the minimum and maximum number of breeders.

### 2.6 Minimum population estimates

The minimum population size as of 2019 included bears identified in 2019, including bears that died in 2019; bears not identified in 2019–2020, but whose presence as of 2019 was inferred by pedigree reconstruction; and bears ≥1 year old, identified not in 2019 but in 2020. The second category included existing bears (i.e., bears identified only before 2019) and hypothetical bears generated by the COLONY software, as described above. In the third category, the age or minimum age of bears was determined based on the year of first genetic identification (i.e., bears identified before 2019 were included), the year when their parent was dead, or on the video data obtained at the time of genetic identification. The combination of a DNA‐based parentage analysis and video data taken at the hair‐trap site sometimes enabled us to determine the age of young bears (i.e., 0–1 years old) that accompanied their mother. Some bears were confirmed to be ≥1 year old when their DNA was collected, and their body sizes were able to be assessed by the video data. Significant differences in body sizes between 0‐ and 1‐year‐old bears enabled us to determine whether bears were ≥1 year old, even if information regarding their age was unavailable. This assessment was made only when we could identify the bear in the video clip with 100% confidence. Therefore, bears that had the possibility of being cub‐of‐the‐year were not included in the minimum population size as of 2019.

## 3 RESULTS

The number of samples analyzed and the number of bears identified by an intensive survey during 2019–2020 are shown in Table 1. By use of tree‐rub traps, 291 and 324 unique individuals were identified in 2019 and 2020, respectively. Among them, nine bears (3.0% of total bears detected by tree‐rub traps; six females and three males) and 17 bears (5.2% of total bears detected by tree‐rub traps; three females and 14 males) were detected at both sides (i.e., east and west side) of the peninsula. The distribution of feces that was successfully analyzed is shown in Figure 2. From the 2‐year intensive genetic survey in 2019–2020, 499 unique bears (281 females and 218 males) were identified. Among them, 172 bears (96 females and 76 males) had been genetically identified by 2018. Finally, with the samples collected between 1998 and 2020, we genotyped 1288 bears (616 females and 672 males), including 1221 bears from the Shiretoko Peninsula (i.e., Shari, Rausu, and Shibetsu towns), and 67 bears from areas adjacent to the peninsula (i.e., Kiyosato and Nakashibetsu towns). The values for genetic diversity measures for 21 microsatellite loci (shown in Appendix A) were nearly identical to those in our previous study, for example, the mean number of alleles were 5.6 (Shimozuru et al., 2019) and 5.8 in the current study. Approximately 58% of the sampled bears (748 bears) were confirmed to be dead, due to management culls, hunting, accidents, or natural causes. All bears were genotyped at all of the loci. We found seven haplotypes in the mitochondrial analysis, which was similar to the results of previous studies on the same population (Shirane et al., 2018): HB‐02 (*N* = 37), HB‐10 (*N* = 139), HB‐11 (*N* = 703), HB‐12 (*N* = 66), HB‐13 (*N* = 122), HB‐new1 (*N* = 107), and HB‐new2 (*N* = 1); and one heteroplasmic pattern: HB‐10/11 (*N* = 113). For the Y chromosomal haplotype analysis, we found four haplotypes (BR1_02, BR1_04, BR1_05, and BR1_06) that were reported in a previous study (Hirata et al., 2017). In addition, based on two markers, UarY369.4 and 15020.1, which were excluded in the above study due to the pseudoheterozygous genotypes identified in bears sampled outside Hokkaido, the haplotypes BR1_04 and BR1_05 were classified into two and three sub‐haplotypes, respectively. Finally, we found seven haplotypes, BR1_02 (*N* = 32), BR1_04a (*N* = 1), BR1_04b (*N* = 339), BR1_05a (*N* = 57), BR1_05b (*N* = 91), BR1_05c (*N* = 1), and BR1_06 (*N* = 149). Two samples were not available for Y chromosomal haplotypes due to an unstable amplification.

**TABLE 1:** Number of samples and bears identified in the Shiretoko peninsula, Hokkaido, Japan, during 2019–2020.

Samples	Feces		Hairs		Other sources^a^		Dead^b^		Total		
Year	2019	2020	2019	2020	2019	2020	2019	2020	2019	2020	2019–2020
No. samples analyzed	459	439	3952	7142	9	29	48	17	—	—	—
No. successful analysis	242	331	2431	3301	9	20	48	17	—	—	—
No. unique bears	111	138	293	335	9	16	48	17	355^c^	373^c^	499^c^
female/male	57/54	72/66	176/117	192/143	5/4	10/6	14/34	5/12	203^c^/152	214^c^/159	281^c^/218

^a^ Other methods; biopsy‐darting, blood stains, and saliva.

^b^ Dead; management kills, hunting, and natural death.

^c^ The number included one and three visually identified, not genetically identified individuals in 2019 and 2020, respectively.

Table 2 summarizes the results of the parentage analysis with CERVUS. Among the 499 unique bears identified in 2019–2020, seven males had the HB‐02 mitochondrial haplotype. This haplotype was common in the middle part of Hokkaido, and females with this haplotype occurred roughly 80 km west or south of the peninsula (Matsuhashi et al., 1999). However, in our previous (Shirane et al., 2018) and present studies, no females with HB‐02 were identified throughout the peninsula among 341 and 591 females, respectively, which made it reasonable to consider that they had immigrated from outside the peninsula. Therefore, those males were excluded from the candidate bears that were possibly born inside the peninsula. Both parents were assigned for over two‐thirds of the remaining 492 bears, and less than 8% of the bears were unassigned to one parent. Among the 499 bears, including the seven immigrant males, 125 females and 65 males were confirmed to be breeders, due to the existence of ≥1 offspring between 1998 and 2020. In addition, 27 females and 18 males were identified as ≥4 years old as of 2019, based on the year of first visual/genetic identification (12 females and six males), the year of death of their parent (15 females and eight males), or an age estimation at death by counting the cementum annuli of the teeth (four dead males in 2019–2020), although they did not have any breeding record. Among the 499 bears, no bears were assigned as daughters/sons, or mothers/fathers of bears sampled outside the peninsula (i.e., Kiyosato and Nakashibetsu towns). Taken together, among the 499 bears identified in 2019–2020, 152 females and 83 males were confirmed to be adults (i.e., bears with reproductive experience or ≥4 years old) as of 2019.

**TABLE 2:** Key characteristics of the parentage analysis by CERVUS showing the number of breeders and adults in the Shiretoko peninsula, Hokkaido, Japan, in 2019–2020.

	2019–2020	
	Number	%
Bears identified in 2019–2020	499^a^	—
Females	281	56.3
Males	218^a^	43.7
Triads	330	67.1
Dyads		
With mother	47	9.6
With father	76	15.4
With “no parent”	39	7.9
Female breeders^b^	125	44.4^c^
Male breeders^b^	65^d^	29.8^c^
Ratio of dams: sires	2.04	—
Females ≥4 years, no breeding record	27	9.6^c^
Males ≥4 years, no breeding record	18	8.3^c^
No. breeders or ≥4 years old (Females/Males)	235 (152/83)	47.1 (54.1^c^/38.1^c^)

^a^ Seven males, originated out of the peninsula, were included, but excluded as potential offspring in the parentage analysis.

^b^ Individuals with at least one offspring between 1998 and 2020.

^c^ Percentage among same sex.

^d^ Four males, originated outside the peninsula, were included.

Table 3 summarizes estimations of the breeding population by including past‐identified breeders (previously existed, but not identified in 2019–2020) and hypothetical parents, based on a pedigree reconstruction by the combination of CERVUS and COLONY analyses. Among the bears identified between 1998 and 2018 but not in 2019–2020, 16 females and 10 males (identified between 2008 and 2018) were assigned as parents of bears identified in 2019–2020. Among them, one female was assigned as a mother of a bear that was born in 2018 and was dead in 2020. She was included in the minimum breeding population because it was reasonable to assume that she survived until the time of separation with the offspring in 2019. On the contrary, three females were estimated to be ≥30 years old based on the reconstructed pedigree. By excluding these bears, 9.3 and 5.3 bears were included in the maximum breeding population as of 2019 (Table 3). Subsequently, COLONY generated 51 hypothetical mothers and 37 hypothetical fathers as potential parents of the bears (identified in 2019–2020) that remained unassigned to both or either of the parents in the CERVUS analysis. Among them, two females and one male were included in the minimum breeding population because they were assigned as parents of bears born in 2019 (two hypothetical females) and in 2020 (one hypothetical male). Among the remaining hypothetical parents, 13 females and 16 males were excluded due to the estimated age (two females and three males were estimated to be ≥30 and ≥ 28 years old, respectively), and due to the limitation of maximum maternal/paternal generations (nine females and 13 males were considered to be great‐great‐grandmothers and great‐grandfathers, respectively). In addition, two females were assumed to be dead because they were mothers of resident adult females in the Rusha area, but were not observed in the past 12 years. Finally, the minimum/maximum adult populations of females and males were estimated to be 155–200 and 84–109, respectively.

**TABLE 3:** Population size of breeders and adults estimated by a pedigree reconstruction in the Shiretoko peninsula, Hokkaido, Japan, as of 2019.

	Female	Male
No. breeders (existed)	16	10
Estimated as dead	4	0
Counted as minimum number	1	0
Counted as maximum number	9.3	5.3
No. hypothetical parents	51	37
Estimated as dead	13	16
Counted as minimum number	2	1
Counted as maximum number	36	20
Minimum No. of parents	3	1
Maximum No. of parents	45	25
Breeding population size	128–173	66–91
Adult (≥4 years) population size	155–200	84–109

**FIGURE 3:**
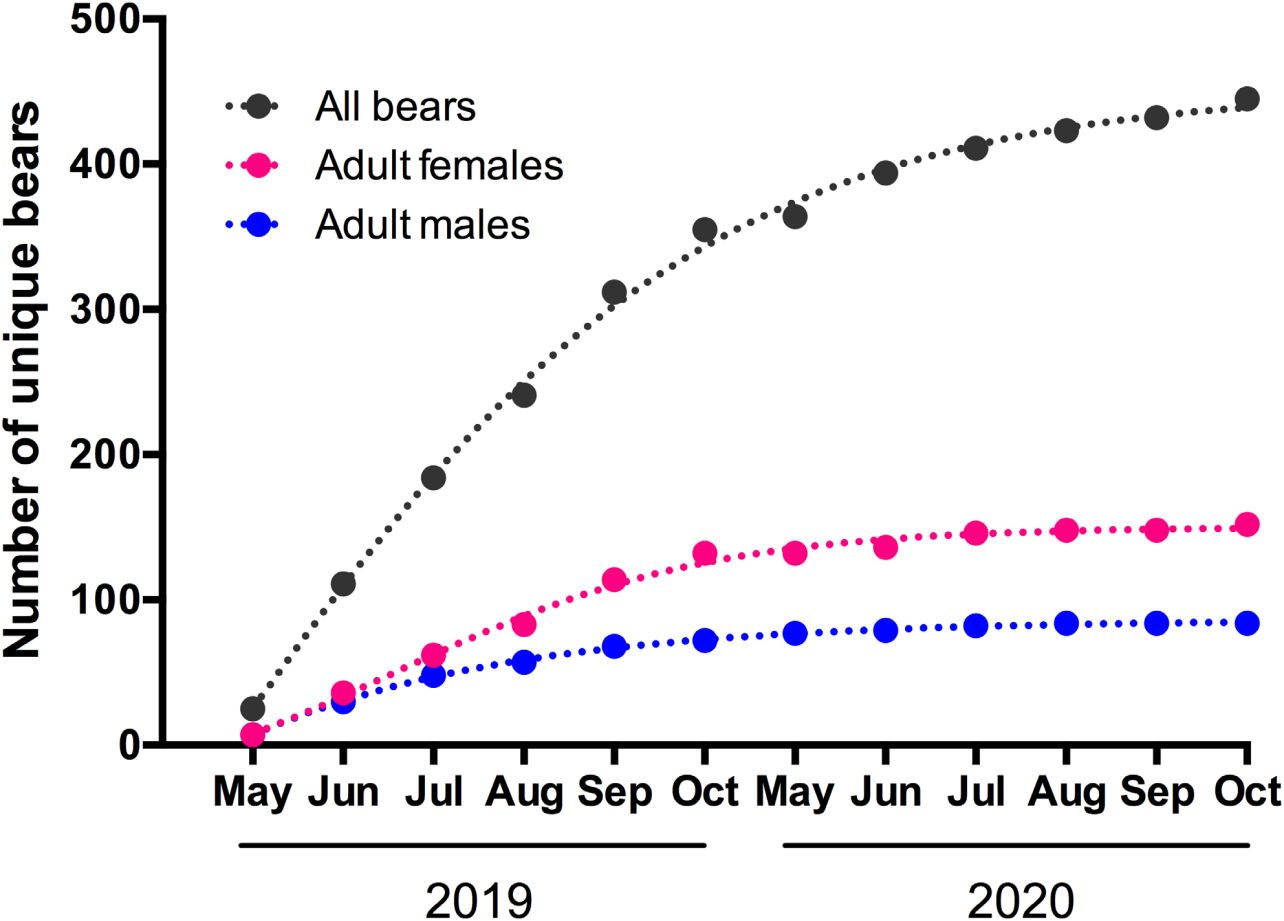
Changes in the cumulative number of unique bears counted as the minimum population in the Shiretoko peninsula, Hokkaido, Japan, in 2019. Bears identified visually or inferred by pedigree reconstruction were excluded from this analysis. Three females were counted as adults, not at the timing of first genetic identification, but when they were proven to be an adult (e.g., at a time when they were confirmed to be present with offspring).

The minimum bear population in 2019 in the Shiretoko Peninsula is shown in Table 4. It was found that a total of 449 (252 females and 197 males) existed as of 2019 in the Shiretoko Peninsula. Changes in the cumulative number of unique bears counted as the minimum population in 2019 are shown in Figure 3. Bears identified visually (one female) or inferred by pedigree reconstruction (one existing female, two hypothetical females, and one hypothetical male) were excluded from this analysis. Three females were counted as adults, not at the timing of first genetic identification, but when they were proven to be an adult (e.g., at a time when they were confirmed to be present with offspring).

**TABLE 4:** Minimum population size in the Shiretoko peninsula, Hokkaido, Japan, as of 2019.

Age	Minimum no. bears in 2019		Bears identified in 2019		Bears identified in 2020	
	Females	Males	Females	Males	Females	Males
≧4	155^a^	85^a^	134^b^ (5)	72 (12)	112 (1)	67 (5)
2–3	14	17	13 (5)	16 (12)	12 (1)	6 (3)
1	4	14	4 (1)	11 (7)	21	27 (4)
0	30	34	21 (3)	25 (2)	32 (2^d^)	18
Unknown	49	47	31	28 (1^d^)	37 (1)	41
Subtotal	252	197	203^b^ (14)	152 (34^d^)	214^b^ (5^d^)	159 (12)
Total	449^b^ ^,^ ^e^		355^b^ (48^d^)		373^c^ (17^d^)	

*Note*: Number in the parenthesis indicates the number of bears died in the given year.

^a^ Three females and one male that were not identified in 2019–2020 but inferred by pedigree reconstruction were included.

^b^ One and ^c^three visually identified bears were included.

^d^ One bear (age unknown) and one cub that died due to natural causes were included in 2019 and 2020, respectively.

^e^ Fifty bears born and four bears possibly born in 2020 were excluded.

## 4 DISCUSSION

We applied a pedigree reconstruction approach to estimate the breeding and adult population size of brown bears on the Shiretoko Peninsula, Japan. Large‐scale, intensive genetic sampling enabled a high rate of parentage assignment, which allowed us to estimate the minimum size of the breeder/adult populations. The adults (≥4 years old as of 2019) accounted for 47.1% of the total unique bears identified in 2019–2020, which was comparable with the percentage of adults (43.0%; ≥5 years old, defined in Craighead et al., 1995) in Yellowstone bears monitored at Trout Creek, 1960–1968, and that in the Swan Mountains, Montana, 1987–1996 (48.2%; ≥5 years old; Mace & Waller, 1998). This suggests that the current method is effective enough to detect breeders/adults among bears without information on age. The estimated breeding/adult population size, although that was the minimum value, was higher than among other brown bear populations in the world, suggesting that this population, which inhabits a small area, has a very high reproductive potential (Schwartz, Miller et al., 2003). Kohira et al. (2009) estimated the population growth rate to be >1 under the conditions where ≥81 adult females ≥5 years old (among 150 females in total) existed in the Shiretoko Peninsula, excluding Shibetsu Town (which accounts for 31% of the total forest cover in the current study area), with eight adult female mortalities/year (7.2 adult [≥5 years old] female mortalities/year in the same area during 2011–2020). Our results suggest that the current harvest rates are below the sustainable level; however, careful attention is still required because some of the parameters used in Kohira et al. (2009) were extrapolated from data obtained from other brown bear populations.

To estimate the maximum breeding population, we made an assumption that the breeding population would not exceed the total number of parents that produced bears identified in 2019–2020. This assumption would be unreliable if the sampling efforts were insufficient or if the sampling area was too limited. In these circumstances, the maximum population size would be severely underestimated. Although most of the hair‐trap sites were placed in coastal areas for ease of access, the combination of hair‐trapping and scat collection enabled intensive genetic sampling in the current study, which was supported by the accumulative curve of unique bears shown in Figure 3. In fact, we identified more than 80% (449 of 559 bears) of the populations estimated by a harvest‐based method (Ministry of the Environment, Government of Japan, 2017), suggesting that the current sampling efforts were sufficient to make such assumptions. Brown bears in this population range from high elevations (e.g., to eat alpine stone pine cones in summer) to coastal areas (e.g., to eat salmon in autumn) depending on seasonal changes in food availability (Shirane et al., 2021). In late summer to autumn, scat samples collected in the Rusha area along the coastline contained large amounts of pine nuts (August), salmon (August–October), and acorns (*Q. crispula*; September–October), suggesting that the bears frequently travel between subalpine or forest areas and coastal areas. Furthermore, the number of bears recorded by automatic cameras installed at tree‐rub traps doubled in September–October in comparison with June–July, suggesting that bears aggregate in the coastal area in the salmon running season (Kawamura et al., 2022). Therefore, it is reasonable to suggest that few bears remained in the area with low sampling intensity all year round (i.e., highly elevated mountain areas), and that most of the bears on the peninsula had the potential to be sampled. In addition, one of the advantages of the current method is that it was possible to infer the presence of the parents without sampling if their offspring were sampled. Male bears disperse from their birthplace at around three years of age (Shirane et al., 2019), which allowed mothers living in the area with a low sampling probability to be detected by the pedigree reconstruction. Therefore, it seems unlikely that the true breeding population exceeded the current estimation. However, we cannot exclude the possibility that we missed a certain number of bears that inhabited the areas with a low sampling intensity, which may have led to underestimation. Further study is needed to clarify the percentage of the true population that was not detected due to the biased sampling locations.

In addition to the possibility of underestimation, it is also necessary to pay careful attention to the possibility of overestimation of the maximum breeding population. One of the disadvantages of this method is that with an increase in the number of bears whose parent(s) are unknown, the number of hypothetical parents increases, which raises the ceiling of the estimate. This concern was partially mitigated by the use of COLONY software, which allowed each hypothetical parent to be assigned to multiple bears based on the promiscuous mating ecology of bears (Steyaert et al., 2012). However, because it is not always possible to know whether they are alive or dead, this leads to an overestimation, particularly in short‐term surveys, as discussed in Creel and Rosenblatt (2013) and Spitzer et al. (2016), in which a population estimation was conducted based on a similar method. In the present study, more than two‐thirds and over 90% of the bears were assigned for both parents and either parent, respectively. This rate of parentage assignment is high compared with other studies targeting brown bears (Norman & Spong, 2015; Sawaya et al., 2014; Spitzer et al., 2016) and other bear species (Zeyl et al., 2009), which allowed us to reduce the generation of hypothetical parents in this study.

This “alive or dead problem” holds true not only for hypothetical parents but also for existing ones. Although the parentage assignment rate was high, the lack of information regarding their survival also can lead to overestimations. In this study, among the 295 existing parents (170 females and 125 males) assigned as the parents of the 492 unique bears identified in the 2‐year period, 222 bears (113 females and 109 males) had already been identified by 2018, of which 196 (97 females and 99 males) were confirmed to be dead. Due to the strong relationship between park managers and hunters on the peninsula, poaching or hunting without a report are very unlikely to have occurred over the past two decades. Those enabled us to reduce the number of breeders without information on their survival, which in turn reduced the difference between the minimum and maximum breeding populations. This was mainly achieved by the accumulation of over 20 years of genetic information preceding large‐scale sampling events. Furthermore, information on age for dead bears (obtained mainly by an analysis of their teeth) and the date of first identification for living bears were very useful to assign the minimum age, which helped improve the accuracy of estimates of the minimum population size as of 2019. We suggest that the current method based on pedigree reconstruction offers less advantage in terms of estimating breeder/adult population sizes based on genetic data obtained by limited sampling events, but works well for populations where continuous genetic surveys (e.g., secured recovery of bears killed for management purposes or by hunting, and their DNA genotyping, and opportunistic collection of DNA samples of live bears from hairs on the rub tree or from feces) have been conducted in advance.

To assume the mortality of hypothetical parents and bears identified only before 2019, we defined two criteria, that is, a maximum number of generations and maximum age as a breeder. This enabled us to exclude 29% (33/114) of those bears from the maximum population size. The adoption of these criteria was a realistic approach on the basis of previous studies; however, it may be too conservative. For example, the minimum ages of some parents were estimated based on the age of the oldest daughter/son in the offspring list, but it was unlikely that the daughter/son was the first offspring that they raised successfully. In fact, among bears included in the maximum breeding number (*N* = 49 and 28, for females and males, respectively), the minimum age for 16 females and 11 males was estimated to be 20 years of age or older, but their real ages may have exceeded the threshold criteria as a breeder. In addition, opportunistic hair‐trapping and scat collection has been conducted throughout the peninsula over the last decade; thus, those older bears should have had a higher possibility of being sampled. Therefore, it is reasonable to think that the maximum breeding size still included a certain number of bears that were already dead. This suggests that the true breeding population size was closer to the minimum than maximum number, which is supported by the accumulative curve of unique adult bears that almost reached a plateau at the end of the 2‐year period.

The sex ratio of breeders was more than twofold (2.04) biased in favor of females, which is unusual compared with other brown bear populations (e.g., 1.20–1.30 in Swedish population; Spitzer et al., 2016). It is generally accepted that there are no sex biases at birth, but the adult sex ratio is more or less biased toward females in most brown bear populations (Schwartz, Miller, et al., 2003). The first factor that may be responsible for the biased sex ratios in breeders is the lower survival rate among males (reviewed in Haroldson et al., 2021), due to greater vulnerability of male bears to human‐caused mortality, e.g., hunting (Bischof et al., 2009). Especially, young males are most vulnerable to human‐caused mortality (McLellan et al., 1999); this was partially supported by the male‐biased probability of human‐caused death in this population, particularly for 2‐ to 3‐year‐old bears when males initiate natal dispersal (Kohira et al., 2009; Shimozuru, Shirane, Yamanaka, et al., 2020). In addition, the high mortality rate in males due to natural causes, for example, starvation due to the high‐energy demand during development in males (predicted by Mattson & Reid, 1991) or intraspecific killing (Schwartz, Miller, et al., 2003), may have accelerated this tendency, although the sex differences in the natural survival rate are still unknown in this population. Another possible factor may be related to sex differences in reproductive opportunities; male reproduction is competitive (Steyaert et al., 2012), and breeding opportunities tend to be biased toward physically mature males, which reduces the possibility for young males with limited breeding experience to be assigned as a father in a parentage analysis. This is consistent with a previous report showing that the frequency of breeding was low in 5‐ to 9‐year old males but high in 10‐ to 14‐year old bears in the Rusha area of the Shiretoko Peninsula (Shimozuru, Shirane, Jimbo, et al., 2020). It is still possible that the bias was partly due to differences in the probability of sampling, that is, lower sampling probability in males. However, the number of bears of unknown age was not very different (49 females vs. 47 males) in the minimum population. In addition, the number of bears whose father was unknown (47) was fewer than that of bears whose mother was unknown (76), which reduced that possibility. This suggests that the female‐biased breeding population (128 vs. 66) or adult (≥4 year) population (155 vs. 84) was not strongly influenced by procedural matters in the current analysis.

The minimum population size (449 individuals as of 2019) in the study area (total area of three towns: 1760 km^2^; total forest cover in the area: 1378 km^2^) indicated that the Shiretoko Peninsula has one of the highest brown bear populations area in the world. The minimum density, although a rough estimate without confidence limits (25.5 and 32.6 bears/100 km^2^, for total area of three towns and for total forest cover in the area, respectively), was much higher than the estimated brown bear density in the interior populations of Europe (e.g., Swedish population: 0.8–1.2 bears/100 km^2^; Bellemain et al., 2005) and North America (0.4–8.0 bears/100 km^2^; Haroldson et al., 2021; Schwartz, Miller, et al., 2003), and also higher than or comparable with the coastal populations in Alaska (18.4–40.0 bears/100 km^2^; Schwartz, Miller, et al., 2003), where a high‐nutrient diet (e.g., salmon) is available in the hyperphagia period. In this study, genetic sampling conducted in two consecutive years (2019–2020) allowed us to increase the minimum population by 28% compared with the number obtained solely in the first year (2019). This was partially achieved by the minimum age assignment for bears identified for the first time in 2020, based on pedigree reconstruction and also on body size assessment in cases where video data were allowed to specify the donor bear. This suggests that a simple count of the detected genotypes, a very classic method, can still provide practicable data through a combination of long‐term, continuous genetic monitoring (by use of appropriate sets of genetic markers) for dead/alive bears and a subsequent multi‐year large‐scale sampling event. However, it is still necessary to pay careful attention to the influence of the movement of bears between inside and outside the peninsula in the present results. Although a previous study showed that bears in Shiretoko Peninsula comprised a single population (Itoh et al., 2013), there are some immigrant males in the lower peninsula (Shirane et al., 2018). We cannot exclude the possibility that large home ranges of males and their seasonal/annual movement may have led to overestimation of the minimum value. Therefore, in the near future, it will be necessary to ascertain how close the minimum value is to the true population size through the use of more sophisticated statistical methods, for example, SECR approaches. A precise estimation of the minimum population size provides applicable and conservative information for wildlife management and conservation, and can be a useful indicator to select the best‐fit model (Solberg et al., 2006), thereby helping to refine population estimates.

In conclusion, our study suggests that pedigree reconstruction is a very useful tool for estimating breeding/adult populations and minimum population size in elusive wildlife species, although the current estimates of maximum breeding/adult populations should be treated with caution. This approach is also applicable to wildlife populations under circumstances where population estimation using statistical models, for example, the SECR approach, is difficult for various reasons, for example, geographical limitations and the behavioral characteristics of study animals. It should be emphasized that not only the sampling intensity for large‐scale sampling events but also the preceding accumulation of information on the genotypes and ages of dead individuals are essential to maximize the utility of this approach. The current study indicates how important an accurate knowledge of animal mortality (due to management culls, hunting, accidents, poaching, and natural deaths) and secured recovery of samples are for monitoring populations of wildlife. A large‐scale, intensive genetic survey is very costly, and therefore, it is not often conducted. In preparation for the opportunity of such surveys, continuous genetic monitoring efforts are needed to maximize the amount and quality of the information regarding demographic parameters.

## AUTHOR CONTRIBUTIONS

**Michito Shimozuru:** Conceptualization (lead); data curation (lead); formal analysis (lead); funding acquisition (lead); investigation (equal); methodology (lead); project administration (lead); visualization (lead); writing – original draft (lead). **Mina Jimbo:** Data curation (equal); investigation (equal); visualization (equal); writing – review and editing (equal). **Keisuke Adachi:** Data curation (equal); formal analysis (supporting); investigation (equal); writing – review and editing (equal). **Kei Kawamura:** Data curation (equal); investigation (equal); writing – review and editing (equal). **Yuri Shirane:** Data curation (equal); investigation (equal); writing – review and editing (equal). **Yoshihiro Umemura:** Data curation (equal); investigation (equal); resources (equal); writing – review and editing (equal). **Tsuyoshi Ishinazaka:** Data curation (equal); investigation (equal); project administration (equal); resources (equal); writing – review and editing (equal). **Masanao Nakanishi:** Data curation (equal); investigation (equal); resources (equal); writing – review and editing (equal). **Mayu Kiyonari:** Data curation (equal); investigation (equal); resources (equal); writing – review and editing (equal). **Masami Yamanaka:** Data curation (equal); funding acquisition (lead); investigation (equal); resources (equal); writing – review and editing (equal). **Yukihiro Amagai:** Data curation (equal); investigation (equal); resources (equal); writing – review and editing (equal). **Ayaho Ijuin:** Data curation (equal); investigation (equal); resources (equal); writing – review and editing (equal). **Tomoki Sakiyama:** Data curation (equal); investigation (equal); resources (equal); writing – review and editing (equal). **Shinsuke Kasai:** Data curation (equal); investigation (equal); resources (equal); writing – review and editing (equal). **Takane Nose:** Data curation (equal); investigation (equal); resources (equal); writing – review and editing (equal). **Masataka Shirayanagi:** Data curation (equal); investigation (equal); resources (equal); writing – review and editing (equal). **Hifumi Tsuruga:** Data curation (equal); investigation (equal); resources (equal); writing – review and editing (equal). **Tsutomu Mano:** Data curation (equal); investigation (equal); resources (equal); writing – review and editing (equal). **Toshio Tsubota:** Supervision (equal); writing – review and editing (equal). **Keita Fukasawa:** Supervision (equal); writing – review and editing (equal). **Hiroyuki Uno:** Funding acquisition (lead); project administration (equal); writing – review and editing (equal).

## CONFLICT OF INTEREST

The authors declare no competing interests.

## SUPPORTING INFORMATION

Has any Supporting Information for Publication been submitted?: Yes

## Data Availability

The data (microsatellite genotypes, mitochondrial haplotypes, and Y‐chromosomal haplotypes) are available in Dryad (doi:10.5061/dryad.z612jm6fk).

## APPENDIX A

### Genetic diversity measures for 21 microsatellite loci for 1288 brown bears in and around the Shiretoko Peninsula, Hokkaido, Japan, in 1998‐2020.

**Table:**

Locus	*N*_A_	*H*_O_	*H*_E_	*PIC*	NE_1p_	NE_pp_	*P*_ID_	*P*_ID‐sib_	F_null_
G1A	7	0.736	0.745	0.707	0.653	0.287	0.103	0.403	0.007
MU05	4	0.691	0.731	0.679	0.695	0.35	0.124	0.416	0.028
MU51	6	0.666	0.68	0.636	0.732	0.368	0.146	0.447	0.011
MU50	7	0.752	0.729	0.684	0.679	0.322	0.118	0.415	‐0.017
G10B	8	0.731	0.779	0.747	0.6	0.234	0.08	0.381	0.032
MU23	8	0.799	0.807	0.781	0.551	0.192	0.063	0.362	0.005
G10X	4	0.594	0.602	0.545	0.806	0.482	0.215	0.503	0.006
G10P	6	0.634	0.618	0.571	0.788	0.44	0.193	0.489	‐0.018
G10C	5	0.712	0.714	0.661	0.712	0.367	0.134	0.427	‐0.001
G10M	6	0.648	0.677	0.628	0.74	0.388	0.153	0.45	0.025
MU09	5	0.582	0.563	0.478	0.837	0.575	0.276	0.538	‐0.018
MU59	6	0.778	0.761	0.721	0.64	0.28	0.097	0.394	‐0.012
MU61	3	0.632	0.631	0.556	0.801	0.509	0.211	0.487	‐0.001
G1D	4	0.442	0.45	0.399	0.899	0.637	0.354	0.614	0.011
MU10	4	0.578	0.566	0.488	0.834	0.56	0.267	0.534	‐0.012
UamD2	5	0.743	0.74	0.692	0.682	0.332	0.116	0.409	‐0.002
UamB5	4	0.617	0.628	0.557	0.803	0.505	0.209	0.488	0.008
MU26	4	0.613	0.636	0.586	0.778	0.434	0.183	0.478	0.018
G10L	9	0.595	0.586	0.552	0.805	0.438	0.205	0.508	‐0.009
G10H	8	0.698	0.709	0.666	0.702	0.338	0.127	0.428	0.008
Cxx20	9	0.832	0.83	0.806	0.516	0.169	0.052	0.348	‐0.001
Mean/									
combined	5.8	0.670	0.675	0.626	1.0×10^−3^	8.6×10^−10^	3.0×10^−18^	5.0×10^−8^	‐0.001
	(Mean)	(Mean)	(Mean)	(Mean)	(Combined)	(Combined)	(Combined)	(Combined)	(Mean)

*F*_null_, predicted frequency of null alleles; *H*_E_, expected heterozygosity; *H*_O_, observed heterozygosity; *N*_A_, number of alleles; NE_1*p*_, non‐exclusion probabilities for one candidate parent; NE_pp_, non‐exclusion probabilities for one candidate parent pair; PIC, polymorphic information content; *p*_ID_, probability of identity; *p*_ID‐sib_, probability of identity for full siblings.
